# The biological function and prognostic significance of ferroptosis-related genes in clear cell renal cell carcinoma

**DOI:** 10.3389/fphar.2025.1515552

**Published:** 2025-04-09

**Authors:** Huizhen Ye, Xiwen Wu, Gehao Liang

**Affiliations:** ^1^ Staff and Faculty Clinic, State Key Laboratory of Oncology in South China, Guangdong Provincial Clinical Research Center for Cancer, Sun Yat-sen University Cancer Center, Guangzhou, China; ^2^ Department of Clinical Nutrition, State Key Laboratory of Oncology in South China, Guangdong Provincial Clinical Research Center for Cancer, Sun Yat-sen University Cancer Center, Guangzhou, China; ^3^ Department of Breast Surgery, State Key Laboratory of Oncology in South China, Guangdong Provincial Clinical Research Center for Cancer, Sun Yat-sen University Cancer Center, Guangzhou, China

**Keywords:** ferroptosis-related genes, prognosis, immunological landscape, single-nucleotide variations, clear cell renal cell carcinoma

## Abstract

**Background:**

Ferroptosis-related genes are essential in cancer development. However, the specific roles of ferroptosis-related genes in clear cell renal cell carcinoma (ccRCC) remain largely unexplored. This study aims to elucidate the biological functions and potential therapeutic implications of ferroptosis-related genes in ccRCC.

**Methods:**

A model integrating the Cancer Genome Atlas (TCGA) database and the GEO dataset was established based on ferroptosis-related genes and clinical data. To determine the proliferative function of ferroptosis-related genes in ccRCC cells, CCK-8 kit assays and colony formation experiments were conducted. Additionally, wound-healing experiments were performed to evaluate the migratory capabilities of these cells.

**Results:**

We identified eight ferroptosis-related genes that are significantly associated with the prognosis of ccRCC patients. The expression levels of these genes exhibited marked differences between tumor tissues and normal tissues, and they were shown to influence both the proliferation and metastasis of ccRCC cells. Subsequently, a model combining ferroptosis-related genes model constructed with gene data and clinical characteristics was constructed, and classified the patients into high- and low-risk groups. The area under the curve (AUC) for this model in diagnosing ccRCC was 0.937. In terms of survival prediction, the AUC values were 0.875, 0.818, and 0.790 at 1, 3, and 5 years, respectively. Notably, high-risk patients exhibited significantly poorer survival outcomes compared to those in the low-risk group. Furthermore, high-risk individuals demonstrated elevated expression of immune checkpoint genes and enhanced antitumor immunity, suggesting that these patients may benefit from immunotherapy.

**Conclusion:**

Ferroptosis-related genes play a critical role in the biological functions of ccRCC cells. Our prognostic model has the potential to be applied in predicting patient outcomes and assessing antitumor immunity in ccRCC.

## 1 Introduction

Renal cell carcinoma (RCC) is a common malignant tumor within the genitourinary system globally. Among its various histological subtypes, clear cell renal cell carcinoma (ccRCC) is the most prominent, accounting for 70%–80% of RCC cases (Sung et al., 2021; Ljungberg et al., 2022). Despite advancements in the diagnosis and treatment of ccRCC, approximately 30% of patients experience metastasis after standard treatment. And accurately predicting prognosis and providing individualized therapy for these patients remains a considerable challenge (Motzer et al., 2013). With the development of sequencing, prognostic signatures based on gene expression or mutation have been shown to be a reliable method for predicting survival outcomes and evaluating treatment responses in cancer patients (Cotta et al., 2023).

Ferroptosis is an iron-dependent form of regulating cell death characterized by oxidative damage, different from other cell death modalities such as pyroptosis, necrosis, apoptosis, and autophagy (Zhang et al., 2023). Ferroptosis is triggered by the accumulation of intracellular iron and lipid peroxides, occurred by dysfunction of lipid metabolism, iron metabolism and antioxidant defense systems (Li J. et al., 2023). Excessive accumulation of both intracellular iron and lipid peroxide induces lipid peroxidation of unsaturated fatty acids in the cellular membrane and finally induces cell death (Yang et al., 2023). Due to its role in the progression of many diseases, such as cardiovascular diseases (Wang and Wu, 2023), neurological diseases (Ryan et al., 2023), and malignant tumors (Hu et al., 2023), ferroptosis has emerged as a hotspot. In studies focused on ccRCC, ferroptosis has been identified as a tumor suppressor, effectively inhibiting tumor growth (Gong et al., 2022), suppressing metastasis and decreasing drug resistance (Chang et al., 2023). Furthermore, the expression levels of ferroptosis-related genes and long non-coding RNAs (lncRNAs) have been demonstrated to serve as prognostic indicators of survival outcomes in patients with ccRCC (Lai et al., 2023; Wei et al., 2022; Shi et al., 2022; Tang et al., 2022). Regardless of the published evidence on the regulation of ferroptosis in ccRCC, the precise mechanisms by which ferroptosis contributes to disease progression and influences targeted therapies for ccRCC remain unclear.

In this study, we identified eight ferroptosis-related genes that regulated ccRCC cell proliferation or migration of ccRCC cells. Utilizing expression data of these genes along with corresponding clinical information, we developed a prognostic signature that can assist in assessing the disease risk in patients. We observed that low-risk patients exhibited better survival outcome, while high-risk patients demonstrated increased infiltration of anti-cancer immune cells, indicating that individuals in the high-risk group may have heightened sensitivity to immunotherapy. In addition, tumors from high-risk patients exhibited a higher frequency of mutations in DNA damage repair genes, such as BAP1 and PRKDC, indicating that these patients may benefit from targeted therapies such as PARP inhibitors and platinum-based chemotherapy.

## 2 Methods

### 2.1 Dataset preparation

Single-nucleotide variations, clinical information, and RNA sequencing data of ccRCC patients, along with normal samples, were obtained from The Cancer Genome Atlas (TCGA) database (https://portal.gdc.cancer.gov/). For the training cohort, data from 517 ccRCC patients from TCGA were included after excluding individuals who had a survival time of zero or missing essential information. A validation cohort comprising 446 patients diagnosed with ccRCC was sourced from the cBioPortal database. After excluding individuals with incomplete or missing data, 439 patients were retained for further analysis. Additionally, for further validation, the cohort of GSE53757 dataset composed of 72 individuals utilizing the GPL570 platform (Affymetrix Human Genome U133 Plus 2.0 Array), were enrolled. To assess the translational expression of relevant genes between tumor and normal tissue, data from the Human Protein Atlas (HPA) online database were also utilized (https://www.proteinatlas.org/) (Uhlén et al., 2015).

### 2.2 Ferroptosis-related gene identification and prognostic ferroptosis-related gene model construction

We collected RNA-sequencing data comprising 537 tumor samples and 72 normal samples from the TCGA database. Utilizing the FerrDb website (http://www.zhounan.org/ferrdb/current/operations/download.html), we identified 844 ferroptosis-related genes. Differential expression and volcano plots of these genes between the tumor and normal tissues were conducted with the “limma” and “ggplot” package. Prognostic ferroptosis-related genes associated with disease progression were identified through univariate and Lasso Cox regression analyses. To normalize the RNA sequencing data, we applied a log2 transformation, after which we constructed gene signatures using the “glmnet” R package. The risk score for this signature was calculated using the following formula: Risk score = ∑ (Expi × βi). The “exp” represents the expression level of each gene and “β” denotes the regression coefficient of each gene.

Incorporating the gene signature score along with main clinical characteristics such as age, gender, stage, and grade, we conducted Cox regression analysis to predict 1-, 3-, and 5-year survival probabilities of this disease. The corresponding score for each factor in model were calculated with R package “nomogramEx.” Subsequently, patients in the training cohort were categorized into two groups based on the median risk score derived from the model.

### 2.3 Cell lines and siRNA transfection

The OSRC2 and CAKI2 cell lines, commonly employed in renal cancer research (Akifumi et al., 2024; Guodong et al., 2013; Hiroshi et al., 2021; Yasutoshi et al., 2012), were obtained from the American Type Culture Collection (ATCC). These cell lines were cultured in RPMI-1640 medium (Gibco, United States) supplemented with 10% fetal bovine serum (FBS) (Gibco, United States) and 1% penicillin/streptomycin (Gibco, United States). All cell cultures were maintained at 37°C in a 5% CO_2_ atmosphere. To knock down specific target genes, small interfering RNA (siRNA) was employed in conjunction with Lipofectamine^®^ 3000 according to the manufacturer’s protocols (Xiwen et al., 2023). Sequences of siRNAs used in this research is shown in Supplementary Table 1. Corresponding primer sequences for the targeted genes are provided in Supplementary Table 2. The gene silencing efficiency of each targeted gene are presented in Supplementary Figure 1 and the raw data is shown in Supplementary Table 3.

### 2.4 Cell proliferation assay

Cells were plated in 96-well plates at a concentration of 2,000 cells per well in 200 μL of growth medium. After incubation for the specified duration, the medium was aspirated, and 20 μL of diluted CCK-8 solution (Dojindo, Japan) was added. The plates were further incubated at 37°C for 1 h, followed by measurement of absorbance at 450 nm (Xiwen et al., 2023). Experiments were performed in three independent groups to ensure consistency and reliability of the results.

### 2.5 Colony formation assays

All cells were seeded in six-well plates at a density of 1,000 cells per well. The culture medium was refreshed every 3 days. After a ten-day incubation period, the medium was aspirated, and the cells were washed twice with PBS. The colonies were then fixed in methanol for 20 min and subsequently stained with 0.1% crystal violet solution for an additional 30 min at room temperature. Digital images of the plates were captured for permanent recording, and colony counting was performed using ImageJ software (version 1.8.0).

### 2.6 Gene set enrichment analysis

Gene set enrichment analysis (GSEA) version 4.1.0 from the Broad Institute (United States) was employed to identify gene sets that exhibited significant differences between high- and low-risk groups. Enrichment was considered significant if the false discovery rate (FDR q-value) was <0.25, |NSE| was <2, and the normal P value was <0.05. Multiple GSEA plots were generated using R packages “plyr,” “grid,” “ggplot2,” and “gridExtra.”

### 2.7 Immune-related analysis

The cellular component, immune microenvironment and cell immune response data were calculated by different algorithms including TIMER (Li et al., 2016), CIBERSORT (Newman et al., 2015), EPIC (Racle et al., 2017), IPS (Charoentong et al., 2017), MCPcounter (Becht et al., 2016) and quantiseq (Finotello et al., 2019) with the R package “IOBR” (Zeng et al., 2021). A heatmap was created to visualize immune differences between the low and high-risk groups. Additionally, data calculated by ssGSEA algorithm were analyzed by multiple packages “GSVA,” “limma” and “GSEABase.” Potential immune check-points (ICBs) were reported in published literature, and the expression levels of these ICBs between the two groups were illustrated using box plots.

### 2.8 Analysis of single-nucleotide variations (SNVs) of ferroptosis-related gene signature

Single-nucleotide variation (SNV) data were obtained from the TCGA database. The genes exhibiting the highest rates of SNV between the two groups were calculated using Microsoft Excel. Waterfall plot was constructed by R package “GenVisR” to display the SNVs associated with our signature.

### 2.9 Statistical analysis

We employed several statistical tools for our analyses, including R software (version 4.0.2.), GraphPad Prism 5 Software and SPSS 22.0. To assess gene intensity via immunohistochemistry based on different staining types (none = 0, light = 1, moderate = 2, deep = 3), we calculated the scores using the formula: ∑ (percentage* intensity). The R package “rms” was utilized to generate a nomogram to predict 1-, 3-, and 5-year survival probability. R packages “survminer” and “survival” were used to draw Kaplan-Meier plotters to evaluate the discrepancy of overall survival (OS) between different subgroups with chi-squared test. Additionally, a Cox proportional hazards analysis presented hazard ratios (HRs) and 95% confidence intervals (CIs) for various clinical characteristics between groups. Time-dependent receiver operating characteristic (ROC) curves for 1-, 3-, and 5-year survival probabilities were analyzed using the R packages “survivalROC” and “timeROC.” The proportions of immune cells, immune microenvironment, immune checkpoint expression levels and several important genes expressions were compared between groups with Wilcoxon test. A significance level of P < 0.05 was applied, with all p values being two-tailed.

## 3 Results

### 3.1 Screening ferroptosis-related genes in the TCGA database and signature construction

**FIGURE 1:**
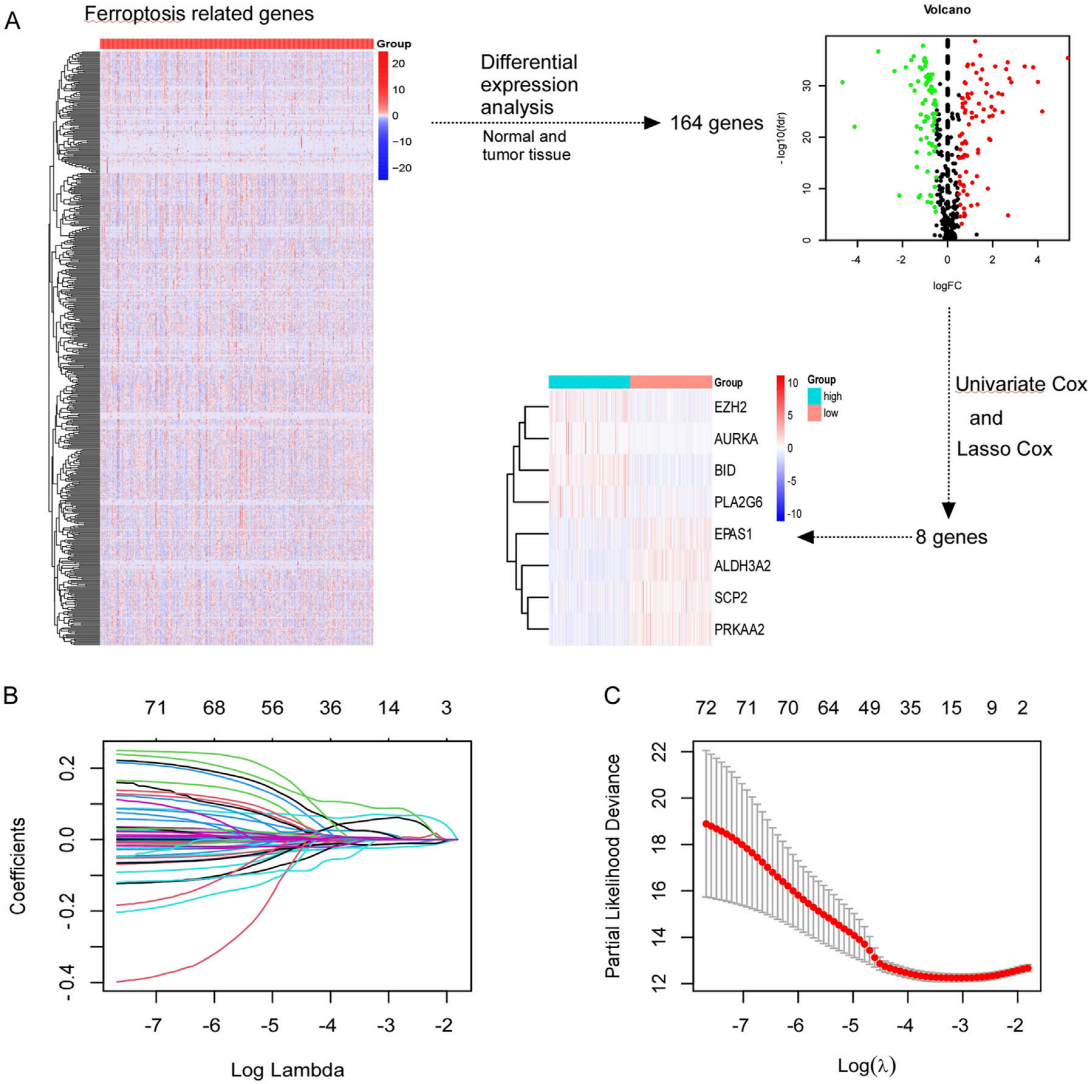
Screening ferroptosis-related genes in TCGA. **(A)** Screening out of eight genes (EZH2, AURKA, BID, PLA2G6, EPAS1, SCP2, PRKAA2, ALDH3A2) which were associated with prognosis of CCRCC patients. **(B, C)** The least absolute shrinkage and selection operator (LASSO) regression performed with the lambda.1se criteria.

In Supplementary Figure 2, we present the study’s flowchart. A total of 844 ferroptosis-related genes were obtained from FerrDb website. Following differential expression analysis between normal and tumor tissue from TCGA database, 164 genes were selected. Through univariate and lasso cox analysis, eight ferroptosis-related genes were selected (EZH2, AURKA, BID, PLA2G6, EPAS1, SCP2, PRKAA2, ALDH3A2), which significantly influence the prognosis of patients with ccRCC (Figure 1A). The filter variable dynamic process diagram (Figure 1B) and cross verification curve (Figure 1C) were performed with the lambda.1 se criteria to show the process of the LASSO regression. The risk score of the ferroptosis-related gene signature was calculated using the following formula: (−0.000045 × EPAS1) + (−0.002464 × SCP2) + (−0.014891 × ALDH3A2) + (−0.010727 × PRKAA2) + (0.061863 × BID) + (0.026166 × AURKA) + (0.012338× EZH2) + (0.029049× PLA2G6).

### 3.2 Translational and transcription expression of ferroptosis-related genes in ccRCC cells

**FIGURE 2:**
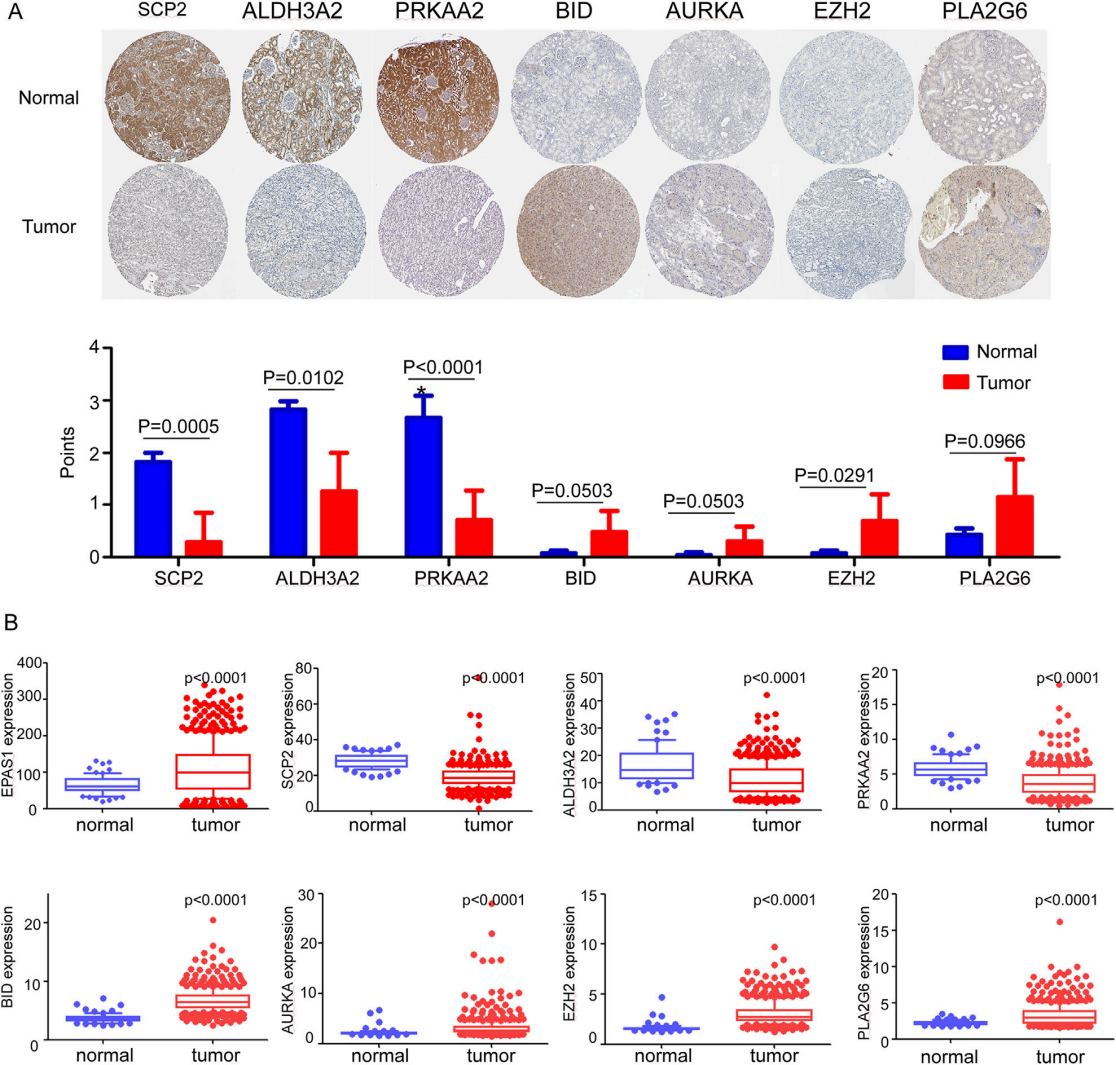
**(A)** Translational expression samples and score of ferroptosis-related genes between tumor and normal tissue in the Human Protein Atlas. **(B)** mRNA expression levels of ferroptosis-related genes between normal and tumor tissue in TCGA dataset.

Initially, the translated data for these ferroptosis-associated genes was obtained from the Human Protein Atlas database (Figure 2A), in which all genes were identified through immunohistochemistry. The genes EZH2 was significantly higher expressed in tumor tissue while SCP2, ALDH3A2, and PRKAA2 were significantly upregulated in normal tissue. Figure 2B illustrates the transcriptional profiles of these eight genes in the TCGA dataset, revealing expression patterns that were consistent with those observed in Figure 2A. In ccRCC tissue, compared to normal kidney tissue, EPAS1, BID, AURKA, EZH2, and PLA2G6 exhibited significantly elevated expression levels. Conversely, the expression of SCP2, ALDH3A2, and PRKAA2 significantly reduced in ccRCC tissue. Supplementary Figure 3 illustrates the transcriptional data for various predictive ferroptosis-related genes from the GSE53757 dataset. The expression results were generally in agreement with those in the TCGA dataset except PLA2G6 gene, which exhibited higher expression in normal kidney tissue.

### 3.3 Biological functions of related genes in RC cells

**FIGURE 3:**
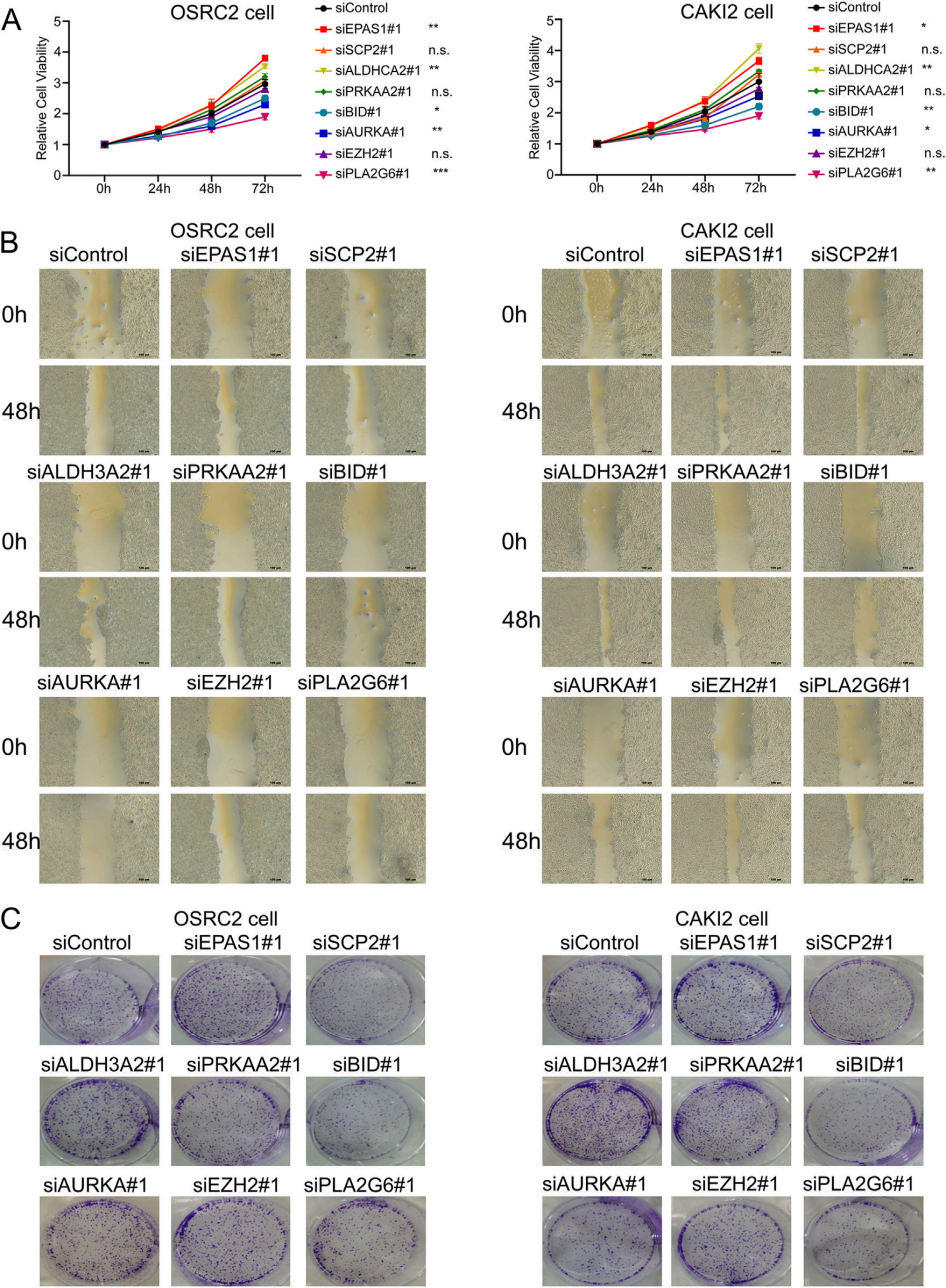
Biological functions of ferroptosis-related genes in ccRCC cells. **(A)** CCK-8 kit assay showed that OSRC2 and CAKI2 cells viability increased after silencing EPAS1 and ALDH3A2, while decreased after silencing BID, AURKA and PLA2G6. **(B)** Wound healing assay experiments showed that knocking down EPAS1 and ALDH3A2 led to increase of invasive OSRC2 and CAKI2 cells, while decreased after silencing BID, AURKA and PLA2G6. **(C)** Plate colony formation assay showed that colony formation of OSRC2 and CAKI2 cells increased after silencing EPAS1 and ALDH3A2, while decreased after silencing BID, AURKA and PLA2G6. (Scalebar-100 μm *p < 0.05, **p < 0.01, and ***p < 0.001, n.s no significance, according to Student’s t-test).

We identified the biological functions of eight ferroptosis-related genes in clear cell renal cell carcinoma (ccRCC). The results from the CCK-8 assay demonstrated that silencing EPAS1 and ALDH3A2 resulted in increased cell viability in OSRC2 and CAKI2 cell lines, while silencing BID, AURKA, and PLA2G6 led to a decrease in cell viability (Figure 3A). Additionally, wound healing assays revealed that the knockdown of EPAS1 and ALDH3A2 enhanced the invasive capabilities of OSRC2 and CAKI2 cells, whereas silencing BID, AURKA, and PLA2G6 significantly reduced their invasiveness (Figure 3B). Plate Colony formation assays further indicated that the colony formation ability of OSRC2 and CAKI2 cells increased following the silencing of EPAS1 and ALDH3A2, while a decrease in colony formation was observed after silencing BID, AURKA, and PLA2G6 (Figure 3C; Supplementary Figure 4). These results suggest that ferroptosis-related genes play a significant role in regulating renal cell carcinoma metastasis.

### 3.4 Efficacy of the ferroptosis-related gene signature in diagnosing ccRCC

**FIGURE 4:**
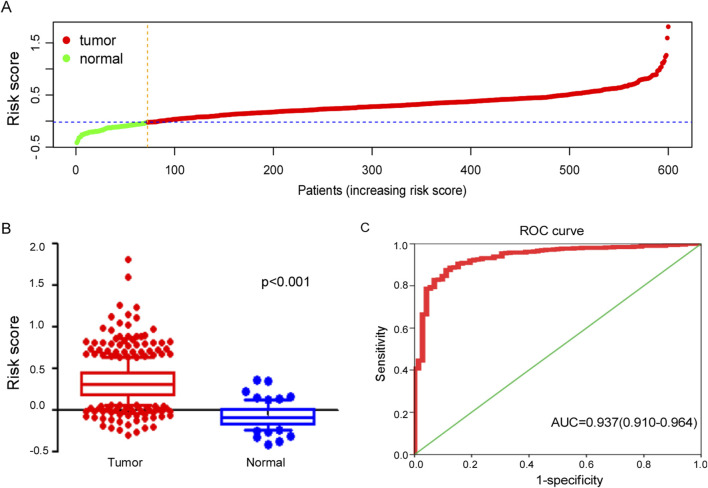
Efficacy of ferroptosis-related genes signature in diagnosing ccRCC. **(A)** Distribution of tumor and normal patients based on the risk score of ferroptosis-related genes signature in TCGA dataset. **(B)** Risk score of signature between tumor and normal samples in TCGA cohort. **(C)** The ROC curve for diagnosing ccRCC with ferroptosis-related genes signature.

The risk score plots derived from the ferroptosis-related gene signature in ccRCC patients and normal individuals were analyzed and presented in Figures 4A, B. These analysis reveals a significant elevation in risk scores for ccRCC when compared to normal kidney tissue, highlighting the potential role of ferroptosis-related genes in the pathogenesis of ccRCC.

The ferroptosis-related gene signature exhibited potential clinical value in diagnosing ccRCC with AUC = 0.937 (95%CI: 0.910–0.964), as illustrated in Figure 4C, which not only highlights the effectiveness of this gene signature in distinguishing ccRCC patients from healthy individuals but also suggests its potential as a valuable biomarker for early diagnosis and treatment planning.

### 3.5 Ferroptosis-related gene model construction

**FIGURE 5:**
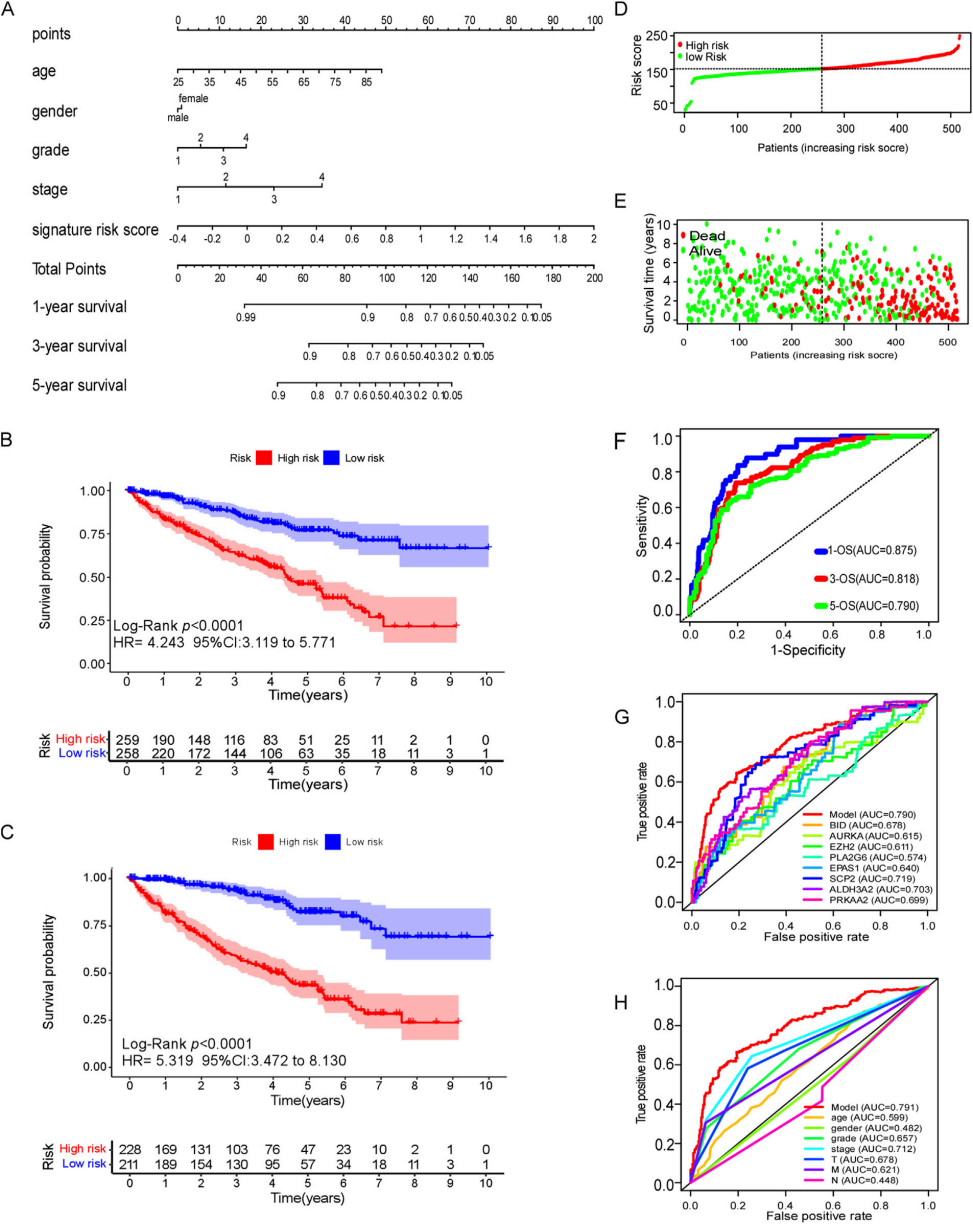
Efficacy of ferroptosis-related genes model in predicting survival risk of ccRCC patients. **(A)** Nomogram of ferroptosis-related genes model to predict the 1, 3, 5-year OS in ccRCC patients. **(B)** Kaplan–Meier survival analysis of CCRCC patients between high-risk groups and low-risk groups in TCGA dataset. **(C)** Kaplan–Meier survival analysis of CCRCC patients between high-risk groups and low-risk groups in cBioPortal database. **(D–E)** Distribution of survival status based on the median risk score of ferroptosis-related genes model in TCGA dataset. **(F)** The AUC of 1,3and 5 years OS by ferroptosis-related genes model. **(G)** The AUC of 5-year OS of every predictive factor of ferroptosis-related genes model. **(H)** The AUC of 5-year OS of ferroptosis-related genes model and certain clinical characteristics.

A ferroptosis-related gene model and nomogram were constructed by multivariate cox regression analysis (Figure 5A), which were composed with ferroptosis-related gene signature score, age, sex, stage and grade of tumor. Age points were calculated with formula: age points = 0.68 * age −16.92. In this model, the point for male patients was set at 0.56, while female patients received 0 points. The points for stages “1, 2, 3, and 4” are as follows: “0.26, 0, 14.85, and 33.29,” respectively, while the points for grades “1, 2, 3, and 4” are “0, 92.07, 96.98, and 100”, respectively. The points for the ferroptosis-related gene signature were computed using the formula: points = 37.94 * signature score + 15.18. These cumulative points were utilized to predict the survival probabilities of patients at 1, 3, and 5 years. Based on the total score, patients were stratified into two groups, with a median total score of 151.84 points serving as the cutoff. This classification was applied consistently in both the training and validation cohorts.

### 3.6 Efficacy of the ferroptosis-related gene model in predicting the prognosis of ccRCC patients

The Kaplan–Meier analysis revealed that high-risk ccRCC patients which was identified by ferroptosis-related gene model, were more likely to experience poor overall survival compared to low-risk individuals in the training cohort (TCGA dataset) (HR 4.243, 95% CI = 3.119–4.243, p < 0.0001) (Figure 5B). Similar findings were observed in the external testing cohort (cBioPortal database) (Figure 5C). The survival status plots, and risk score plots of ferroptosis-related gene model in two groups were displayed in Figures 5D, E, respectively, indicating more death cases as the risk score increase in ccRCC patients, showing a good distinguished power of the model. The area under curve (AUC) values for predicting survival probability at 1, 3, and 5 years were 0.875, 0.818, and 0.790, respectively, in the training cohort (Figure 5F). Notably, the ferroptosis-related gene signature exhibited greater accuracy in predicting the 5-year survival rate of ccRCC patients compared to individual ferroptosis-related genes (Figure 5G). Furthermore, it exhibited superior predictive efficacy for 5-year survival when compared to conventional clinical characteristics such as age, gender, grade, stage and TMN classification (Figure 5H).

### 3.7 Subgroup analysis of the ferroptosis-related gene model in predicting OS

**FIGURE 6:**
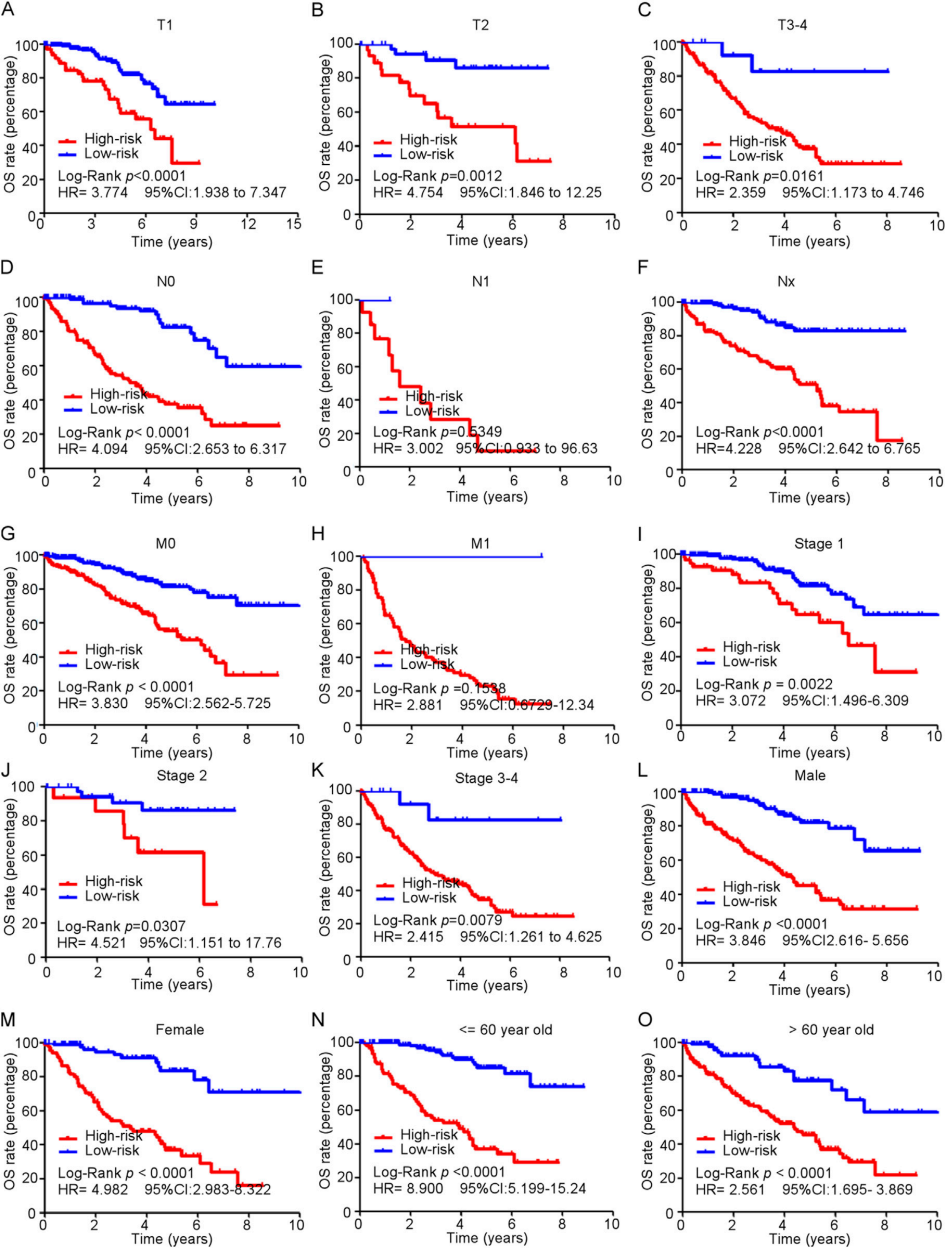
Subgroup analysis of ferroptosis-related genes model in predicting OS in train cohort. **(A–O)** Kaplan-Meier curves for overall survival in TCGA patients in different subgroups.

Subgroup analyses of the ferroptosis-related gene signature were conducted among patients diagnosed with ccRCC to evaluate the model’s performance across various clinical characteristics (Figure 6). These analyses revealed that high-risk patients consistently exhibited poorer overall survival rates compared to low-risk patients across multiple subgroups. This trend was evident in patients categorized by tumor stages T1 to T4 (Figures 6A–C), as well as those classified with N0 (no regional lymph node involvement) and M0 (no distant metastasis) stages (Figures 6D–H). Notably, the model’s predictive accuracy extended to all stages of disease, encompassing stages 1–4 (Figures 6I–K). Moreover, both male and female patients classified as high-risk demonstrated significantly worse survival outcomes compared to low-risk individuals (Figures 6L, M). Additionally, the analysis included different age groups, and the results indicated that high-risk patient experienced diminished overall survival compared to their low-risk peers (Figures 6N, O).

### 3.8 Gene set enrichment analysis (GSEA)

**FIGURE 7:**
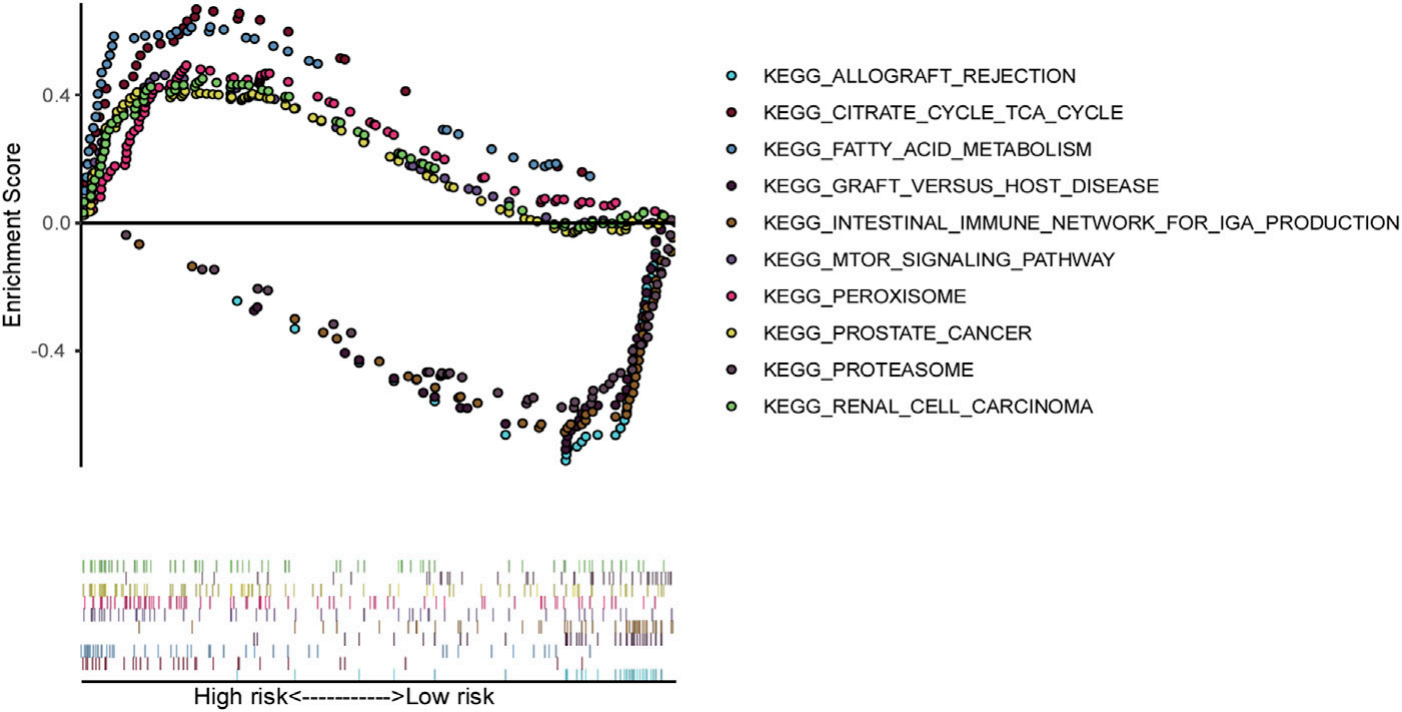
Gene set enrichment analysis (GSEA) based on ferroptosis-related genes model in TCGA ccRCC patients.

Gene set enrichment analysis (GSEA) was employed to investigate the differences in biological profiles between high-risk and low-risk patients with ccRCC. Pathways such as the intestinal immune network for mTOR signaling, fatty acid metabolism, the citrate cycle (TCA cycle), renal cell carcinoma, and peroxisome pathways were enriched in high-risk patients, suggesting the alterations in this pathway may contribute to the aggressive behavior of tumors in high-risk patients. Conversely, the low-risk group displayed enrichment in pathways related to immune response and tissue rejection, including the intestinal immune network for IgA production, allograft rejection, graft versus host disease, and proteasome pathways (Figure 7), which suggest that the low-risk patients may have a better immune response, potentially facilitating better control of tumor growth and progression.

### 3.9 Immunity and gene expression

**FIGURE 8:**
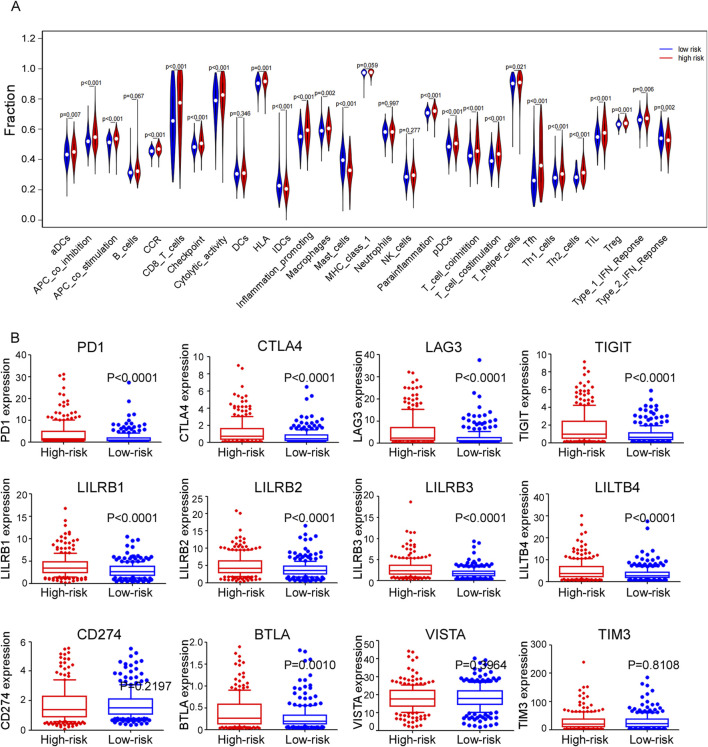
**(A)** Violet plots comparing the expression levels of different immune-related functions of ssGASE dataset between high and low risk groups. **(B)** Expression levels of different immune checkpoints between high and low risk groups in train cohort.

A heatmap was generated using CIBERSORT, EPIC, IPS, MCPcounter, quantiseq and TIMER algorithms to illustrate the immune landscape in high- and low-risk groups (Supplementary Figure 5). Differences in infiltration of 16 types of immune cells between two groups were analyzed using ssGSEA (Figure 8A). Cancer-inhibitory immune cells, including activated dendritic cells (aDCs), CD8^+^ T cells, macrophages, tumor cell infiltrating lymphocytes (TILs) and T regulatory cells (Tregs), were significantly higher in the high-risk group, suggesting a stronger anti-tumor immune response in these patients. Additionally, thirteen immune functional scores, such as CC chemokines (CCR), checkpoints, cytolytic activity, human leukocyte antigen (HLA), and inflammation-promoting factors, showed significant differences between these two groups. Specifically, the expression levels of immune checkpoints such as PD1, CTLA4, LAG3, TIGIT, LILRB, and BTLA were significantly elevated in the high-risk group (Figure 8B). These findings suggest a potential therapeutic advantage from immunotherapy for high-risk patients, highlighting the role of these pathways in cancer progression.

### 3.10 SNV analysis of genes

**FIGURE 9:**
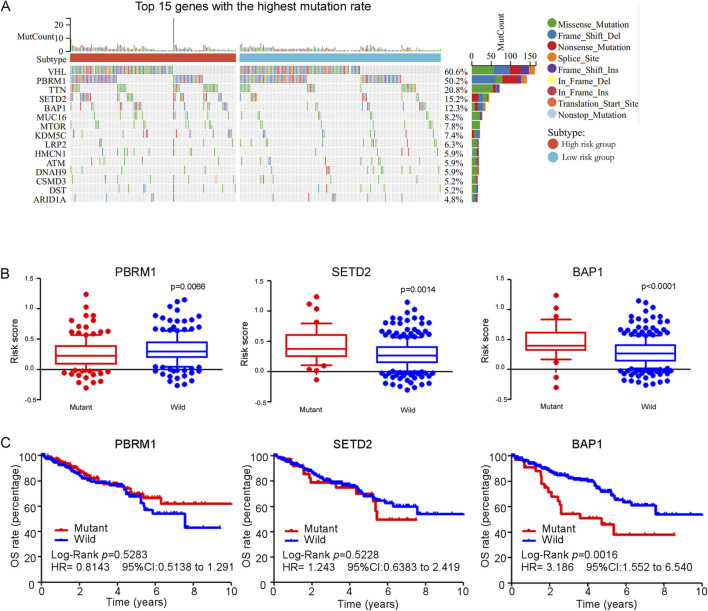
**(A)** Waterfall plot and mutation frequency of genes with SNV expression in high and low risk groups. **(B)** The score of ferroptosis-related genes model between gene (PBRM1, SETD2 and BAP1) mutant group and wild group in TCGA ccRCC patients. **(C)** Kaplan-Meier curves for overall survival in gene (PBRM1, SETD2 and BAP1) mutant group and wild group among TCGA ccRCC patients.

We analyzed all genes exhibiting single nucleotide variant (SNV) mutations, ultimately identifying the 15 genes with the highest mutation rates, as illustrated in Figure 9A. Among these, VHL displayed the highest mutation rate (60.6%), followed by PBRM1 (50.2%), TTN (20.8%), SETD2 (15.2%), BAP1 (12.3%), etc. The prevalence of mutations in these genes showed their potential significance in the pathogenesis of ccRCC. Interestingly, our analysis revealed that patients with mutations in PBRM1 had significant lower risk scores compared to those with a wild-type background. In contrast, mutations in SETD2 and BAP1 were correlated with increased risk scores when compared to their wild-type counterparts (Figure 9B), indicating a potential association with more aggressive disease characteristics and poorer outcomes. Kaplan-Meier analysis revealed that patients with a BAP1 mutation exhibited poorer overall survival (OS) compared to those with wild-type BAP1 (Figure 9C), suggesting that BAP1 mutation may serve as an independent prognostic factor for predicting the outcome of ccRCC patients.

## 4 Discussion

Ferroptosis is a form of iron-dependent cell death characterized by oxidative damage, which is triggered by dysfunction in lipid metabolism, iron metabolism, and antioxidant defense mechanisms. Overaccumulation of intracellular iron and lipid peroxide induces lipid peroxidation of unsaturated fatty acids in the cellular membrane, and ultimately culminate in cell death. In studies on ccRCC, ferroptosis has been identified as a tumor-suppressive mechanism that inhibits tumor growth, suppresses metastasis, and reduces drug resistance. Additionally, certain ferroptosis-related genes or lncRNAs have demonstrated the potential for predicting the survival probability of ccRCC patients. Despite the existing literature on the regulation of ferroptosis in ccRCC, the precise role of ferroptosis in disease progression and its impact on treatment outcomes remains inadequately understood.

We selected eight ferroptosis-related genes (EZH2, AURKA, BID, PLA2G6, EPAS1, ALDH3A2, SCP2, PRKAA2) for prognostic signature construction. A review of existing literature reveals that three of these genes (EZH2, EPAS1, and AURKA) have been demonstrated to promote the proliferation and metastasis of ccRCC (Figg et al., 2023; Hong et al., 2023; Li et al., 2022). In contrast, the remaining five genes (BID, PLA2G6, SCP2, ALDH3A2, and PRKAA2) have also been implicated in influencing the progression of ccRCC. In this study, we re-evaluated the expression levels of these eight ferroptosis-related genes in patients with ccRCC. Our findings revealed that EZH2, AURKA, BID, PLA2G6, and EPAS1 exhibited significantly elevated expression levels in ccRCC tumor tissues compared to normal tissues. Conversely, SCP2, ALDH3A2, and PRKAA2 were found to be expressed at lower levels in tumor tissues. In biological experiments, AURKA, BID, and PLA2G6 functioned as tumor promoters by enhancing the proliferation and migration of ccRCC cells. In contrast, ALDH3A2 exhibited an inhibitory effect on both the proliferation and migration of ccRCC cells. These findings indicate that each selected gene contributes significantly to either the regulation of biological functions or clinical application value. Consequently, the ferroptosis-related gene model we identified are convincing.

Our findings may assist clinicians in the diagnosis and therapeutic decision making of ccRCC. Firstly, the ferroptosis-related gene signatures demonstrated a remarkable ability to distinguish between ccRCC tumor tissue and normal tissue, achieving an AUC of 0.937 (95% CI: 0.910–0.964). Thus, pathologists could detect the expression of these eight genes to differentiate tumor and non-tumor tissue when facing diagnostic difficulties. Second, despite standard treatment, approximately 30% of ccRCC patients suffer recurrence or metastasis (Yue et al., 2021). Our prognostic model was converted into a formula to predict survival probability, providing a visual and convenient method for identifying patients who may require more aggressive treatment or closer follow-up. Third, ferroptosis-related gene model provided the immune landscape of ccRCC tumor tissue. We found that high-risk patients exhibited more anticancer immune cell (i.e., CD8^+^ T Cell) infiltration and higher levels of immune checkpoints molecules (i.e., PD1, CTLA4). These results suggest that high-risk patients may derive greater benefit from immunotherapy, suggesting the potential for targeted therapeutic strategies in this subgroup. Fourth, tumors from high-risk patients displayed a higher frequency of mutations in DNA damage repair genes, such as BAP1 (Kwon et al., 2023) and PRKDC (Chen et al., 2021). Notably, tumors characterized by dysfunction in the DNA damage repair pathway have been shown to exhibit increased sensitivity to PARP inhibitors and platinum-based chemotherapy (Li Q. et al., 2023; Catalano et al., 2022; Yusoh et al., 2020; Basourakos et al., 2017). Therefore, high-risk ccRCC patients may be considered for treatment with PARP inhibitors or platinum-based chemotherapy, particularly in cases where standard treatment options have proven ineffective.

The study also had a few limitations. First, the gene and clinical data information were solely derived from TCGA and GEO databases, without verification from real-world ccRCC tumor tissue. Second, our study highlighted the significance of ferroptosis-related genes solely in predicting OS, without implicating their role in treatment efficacy for ccRCC patients. Third, we evaluated the tumor immunity of high- and low-risk ccRCC patients exclusively using the CIBERSORT algorithm, without validation through immunohistochemical analysis, indicating a need for further experimental studies. Fourth, due to ethical applications for animal experiments and the long duration and large number of animals required, animal experiments cannot be conducted temporarily. Finally, while the selected ferroptosis-related genes were proven to regulate the proliferation or migration of ccRCC cells, the underlying mechanisms was not explored in this study.

## 5 Conclusion

In summary, we successfully constructed a prognostic model for predicting the survival of ccRCC patients based on eight ferroptosis-related genes and clinical characteristics, which not only offer a valuable tool for clinical decision-making, but also provide a foundation for further investigation into ferroptosis as a potential therapeutic target in ccRCC.

## Data Availability

The original contributions presented in the study are included in the article/Supplementary Material, further inquiries can be directed to the corresponding author.

## Supplementary material

SUPPLEMENTARY FIGURE 1 Silencing efficiency of each target gene in clear cell renal cell carcinoma cell lines.

SUPPLEMENTARY FIGURE 2 The flowchart of our study.

SUPPLEMENTARY FIGURE 3 Expression levels of different predictive factor of ferroptosis-related genes signature between normal and tumor tissue in test cohort GSE53737.

SUPPLEMENTARY FIGURE 4 (A) The percentage of wound healing in OSRC2 and CAKI2. **(B)** The number of clones in OSRC2 and CAKI2 cells.

SUPPLEMENTARY FIGURE 5 Heatmap for immune responses based on CIBERSORT, EPIC, IPS, MCPcounter, quantiseq and TIMER algorithms between high and low risk group.

## Abbreviations

ccRCC, clear cell renal carcinoma; GSEA, Gene set enrichment analysis; SNV, Analysis of single-nucleotide variations; TCGA, The Cancer Genome Atlas.

## References

[B1] Akifumi O. Kotaro S. Makoto K. Yumi S. Yasuyuki K. Kei Y. (2024). Extrajunctional CLDN10 cooperates with LAT1 and accelerates clear cell renal cell carcinoma progression. Cell Commun. Signal 22 (1), 588. 10.1186/s12964-024-01964-5 39639312 PMC11619122

[B2] Basourakos S. Li L. Aparicio A. Corn P. Kim J. Thompson T. (2017). Combination platinum-based and DNA damage response-targeting cancer therapy: evolution and future directions. Curr. Med. Chem. 24 (15), 1586–1606. 10.2174/0929867323666161214114948 27978798 PMC5471128

[B3] Becht E. Giraldo N. Lacroix L. Buttard B. Elarouci N. Petitprez F. (2016). Estimating the population abundance of tissue-infiltrating immune and stromal cell populations using gene expression. Genome Biol. 17 (1), 218. 10.1186/s13059-016-1070-5 27765066 PMC5073889

[B4] Catalano F. Borea R. Puglisi S. Boutros A. Gandini A. Cremante M. (2022). Targeting the DNA damage response pathway as a novel therapeutic strategy in colorectal cancer. Cancers 14 (6), 1388. 10.3390/cancers14061388 35326540 PMC8946235

[B5] Chang K. Chen Y. Zhang X. Zhang W. Xu N. Zeng B. (2023). DPP9 stabilizes NRF2 to suppress ferroptosis and induce sorafenib resistance in clear cell renal cell carcinoma. Cancer Res. 83, 3940–3955. 10.1158/0008-5472.CAN-22-4001 37713596

[B6] Charoentong P. Finotello F. Angelova M. Mayer C. Efremova M. Rieder D. (2017). Pan-cancer immunogenomic analyses reveal genotype-immunophenotype relationships and predictors of response to checkpoint blockade. Cell Rep. 18 (1), 248–262. 10.1016/j.celrep.2016.12.019 28052254

[B7] Chen Y. Li Y. Xiong J. Lan B. Wang X. Liu J. (2021). Role of PRKDC in cancer initiation, progression, and treatment. Cancer Cell Int. 21 (1), 563. 10.1186/s12935-021-02229-8 34702253 PMC8547028

[B8] Cotta B. Choueiri T. Cieslik M. Ghatalia P. Mehra R. Morgan T. (2023). Current landscape of genomic biomarkers in clear cell renal cell carcinoma. Eur. Urol. 84 (2), 166–175. 10.1016/j.eururo.2023.04.003 37085424 PMC11175840

[B9] Figg W. Fiorini G. Chowdhury R. Nakashima Y. Tumber A. McDonough M. (2023). Structural basis for binding of the renal carcinoma target hypoxia-inducible factor 2α to prolyl hydroxylase domain 2. Proteins 91, 1510–1524. 10.1002/prot.26541 37449559 PMC10952196

[B10] Finotello F. Mayer C. Plattner C. Laschober G. Rieder D. Hackl H. (2019). Correction to: molecular and pharmacological modulators of the tumor immune contexture revealed by deconvolution of RNA-seq data. Genome Med. 11 (1), 50. 10.1186/s13073-019-0655-5 31358023 PMC6661746

[B11] Gong Q. Guo Z. Sun W. Du X. Jiang Y. Liu F. (2022). CX3CL1 promotes cell sensitivity to ferroptosis and is associated with the tumor microenvironment in clear cell renal cell carcinoma. BMC Cancer 22 (1), 1184. 10.1186/s12885-022-10302-2 36397015 PMC9670481

[B12] Guodong Z. Liang L. Lei L. Qiang D. Wenbin S. Shuyuan Y. (2013). The expression and evaluation of androgen receptor in human renal cell carcinoma. Urology 83 (2), 510.e19–24. 10.1016/j.urology.2013.10.022 24332122

[B13] Hiroshi F. Yuta T. Tomoki T. (2021). Triple-mutated oncolytic herpes virus for treating both fast- and slow-growing tumors. Cancer Sci. 112 (8), 3293–3301. 10.1111/cas.14981 34036669 PMC8353919

[B14] Hong S. Hwang H. Son D. Kim E. Park S. Yoon Y. (2023). Inhibition of EZH2 exerts antitumorigenic effects in renal cell carcinoma via LATS1. FEBS Open Bio 13 (4), 724–735. 10.1002/2211-5463.13579 PMC10068324 36808829

[B15] Hu S. Chu Y. Zhou X. Wang X. (2023). Recent advances of ferroptosis in tumor: from biological function to clinical application. Biomed. and Pharmacother. 166, 115419. 10.1016/j.biopha.2023.115419 37666176

[B16] Kwon J. Lee D. Lee S. (2023). BAP1 as a guardian of genome stability: implications in human cancer. Exp. and Mol. Med. 55 (4), 745–754. 10.1038/s12276-023-00979-1 37009801 PMC10167335

[B17] Lai J. Miao S. Ran L. (2023). Ferroptosis-associated lncRNA prognostic signature predicts prognosis and immune response in clear cell renal cell carcinoma. Sci. Rep. 13 (1), 2114. 10.1038/s41598-023-29305-5 36747047 PMC9902540

[B18] Li B. Severson E. Pignon J. Zhao H. Li T. Novak J. (2016). Comprehensive analyses of tumor immunity: implications for cancer immunotherapy. Genome Biol. 17 (1), 174. 10.1186/s13059-016-1028-7 27549193 PMC4993001

[B19] Li J. Zheng S. Fan Y. Tan K. (2023a). Emerging significance and therapeutic targets of ferroptosis: a potential avenue for human kidney diseases. Cell Death and Dis. 14 (9), 628. 10.1038/s41419-023-06144-w PMC10516929 37739961

[B20] Li P. Chen T. Kuang P. Liu F. Li Z. Liu F. (2022). Aurora-A/FOXO3A/SKP2 axis promotes tumor progression in clear cell renal cell carcinoma and dual-targeting Aurora-A/SKP2 shows synthetic lethality. Cell death and Dis. 13 (7), 606. 10.1038/s41419-022-04973-9 PMC9279325 35831273

[B21] Li Q. Qian W. Zhang Y. Hu L. Chen S. Xia Y. (2023b). A new wave of innovations within the DNA damage response. Signal Transduct. Target. Ther. 8 (1), 338. 10.1038/s41392-023-01548-8 37679326 PMC10485079

[B22] Ljungberg B. Albiges L. Abu-Ghanem Y. Bedke J. Capitanio U. Dabestani S. (2022). European association of urology guidelines on renal cell carcinoma: the 2022 update. Eur. Urol. 82 (4), 399–410. 10.1016/j.eururo.2022.03.006 35346519

[B23] Motzer R. Hutson T. Cella D. Reeves J. Hawkins R. Guo J. (2013). Pazopanib versus sunitinib in metastatic renal-cell carcinoma. N. Engl. J. Med. 369 (8), 722–731. 10.1056/NEJMoa1303989 23964934

[B24] Newman A. Liu C. Green M. Gentles A. Feng W. Xu Y. (2015). Robust enumeration of cell subsets from tissue expression profiles. Nat. methods 12 (5), 453–457. 10.1038/nmeth.3337 25822800 PMC4739640

[B25] Racle J. de Jonge K. Baumgaertner P. Speiser D. Gfeller D. (2017). Simultaneous enumeration of cancer and immune cell types from bulk tumor gene expression data. eLife 6, e26476. 10.7554/eLife.26476 29130882 PMC5718706

[B26] Ryan S. Ugalde C. Rolland A. Skidmore J. Devos D. Hammond T. (2023). Therapeutic inhibition of ferroptosis in neurodegenerative disease. Trends Pharmacol. Sci. 44 (10), 674–688. 10.1016/j.tips.2023.07.007 37657967

[B27] Shi Z. Zheng J. Liang Q. Liu Y. Yang Y. Wang R. (2022). Identification and validation of a novel ferroptotic prognostic genes-based signature of clear cell renal cell carcinoma. Cancers 14 (19), 4690. 10.3390/cancers14194690 36230613 PMC9562262

[B28] Sung H. Ferlay J. Siegel R. Laversanne M. Soerjomataram I. Jemal A. (2021). Global cancer statistics 2020: GLOBOCAN estimates of incidence and mortality worldwide for 36 cancers in 185 countries. CA A Cancer J. Clin. 71 (3), 209–249. 10.3322/caac.21660 33538338

[B29] Tang F. Zhang J. Zhu L. Lai Y. Li Z. Lu Z. (2022). Construction of A Novel ferroptosis-related prognostic risk signature for survival prediction in clear cell renal cell carcinoma patients. Urology J. 19 (4), 289–299. 10.22037/uj.v19i.6999 35598038

[B30] Uhlén M. Fagerberg L. Hallström B. Lindskog C. Oksvold P. Mardinoglu A. (2015). Proteomics. Tissue-based map of the human proteome. Science 347 (6220), 1260419. 10.1126/science.1260419 25613900

[B31] Wang Y. Wu J. (2023). Ferroptosis: a new strategy for cardiovascular disease. Front. Cardiovasc. Med. 10, 1241282. 10.3389/fcvm.2023.1241282 37731525 PMC10507265

[B32] Wei S. Feng B. Bi M. Guo H. Ning S. Cui R. (2022). Construction of a ferroptosis-related signature based on seven lncRNAs for prognosis and immune landscape in clear cell renal cell carcinoma. BMC Med. Genomics 15 (1), 263. 10.1186/s12920-022-01418-2 36528763 PMC9758795

[B33] Xiwen W. Ying Z. Gehao L. Huizhen Y. (2023). Cuproptosis-related lncRNAs potentially predict prognosis and therapy sensitivity of breast cancer. Front. Pharmacol. 14, 1199883. 10.3389/fphar.2023.1199883 37529698 PMC10390311

[B34] Yang L. Liu Y. Zhou S. Feng Q. Lu Y. Liu D. (2023). Novel insight into ferroptosis in kidney diseases. Am. J. Nephrol. 54, 184–199. 10.1159/000530882 37231767

[B35] Yasutoshi Y. Hideo H. Naohiko S. Hirofumi Y. Takeshi Y. Toshihiko I. (2012). Tumor-suppressive microRNA-135a inhibits cancer cell proliferation by targeting the c-MYC oncogene in renal cell carcinoma. Cancer Sci. 104 (3), 304–312. 10.1111/cas.12072 23176581 PMC7657112

[B36] Yue W. Xian W. Huan F. Bintao H. Bo L. Yang L. (2021). Transcriptome analyses identify an RNA binding Protein related prognostic model for clear cell renal cell carcinoma. Front. Genet. 11, 617872. 10.3389/fgene.2020.617872 33488680 PMC7817999

[B37] Yusoh N. Ahmad H. Gill M. (2020). Combining PARP inhibition with platinum, ruthenium or gold complexes for cancer therapy. ChemMedChem. 15 (22), 2121–2135. 10.1002/cmdc.202000391 32812709 PMC7754470

[B38] Zeng D. Ye Z. Shen R. Yu G. Wu J. Xiong Y. (2021). IOBR: multi-omics immuno-oncology biological research to decode tumor microenvironment and signatures. Front. Immunol. 12, 687975. 10.3389/fimmu.2021.687975 34276676 PMC8283787

[B39] Zhang K. He Z. Thakur A. Hu X. Gaurav I. Yang Z. (2023). Editorial: the roles of ion-induced cell death in cancer treatment: volume II. Front. Pharmacol. 14, 1289829. 10.3389/fphar.2023.1289829 37786749 PMC10541952

