# Supplementary figures and images for: The biological function and prognostic significance of ferroptosis-related genes in clear cell renal cell carcinoma
Source: Front Pharmacol. 2025 Apr 9;16:1515552. doi: 10.3389/fphar.2025.1515552 (PMC12014568; doi:10.3389/fphar.2025.1515552)

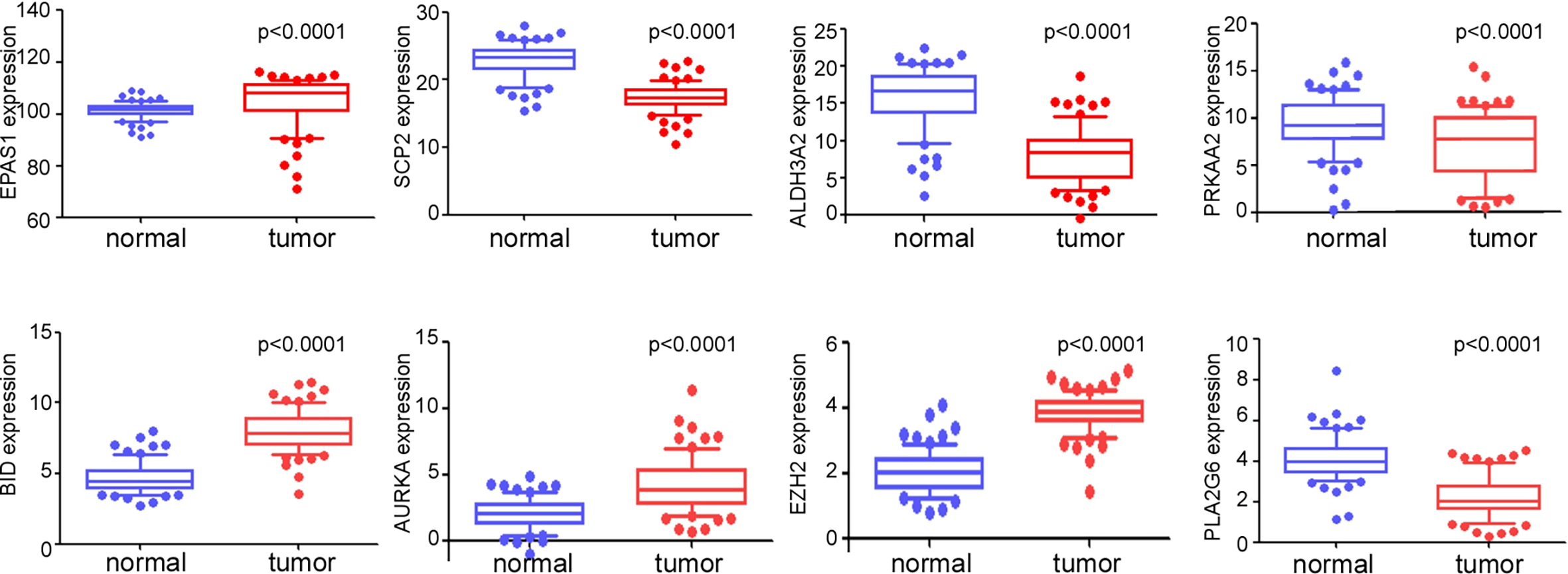

Supplement: Supplementary file 4 [file Image3.tif]

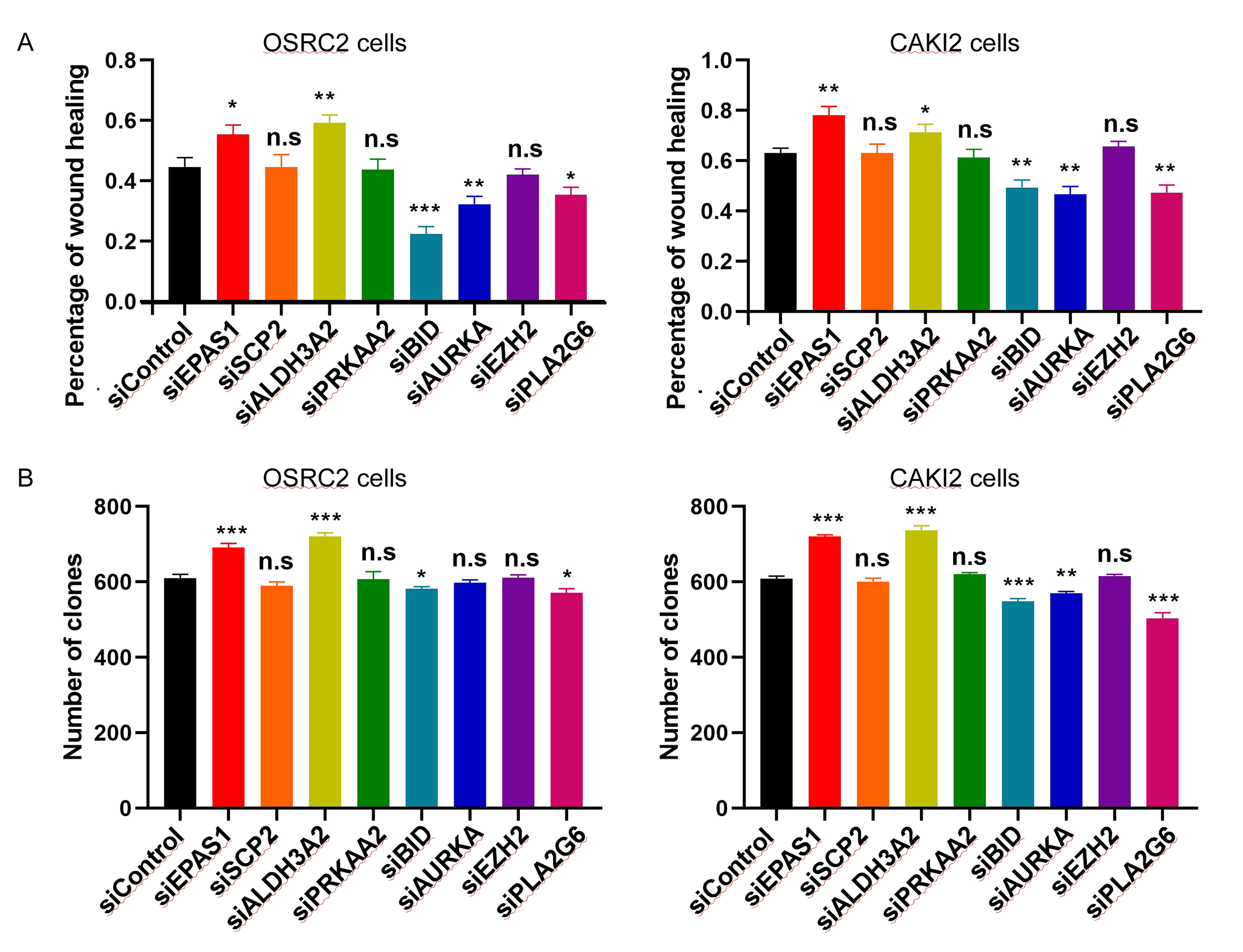

Supplement: Supplementary file 5 [file Image4.tif]

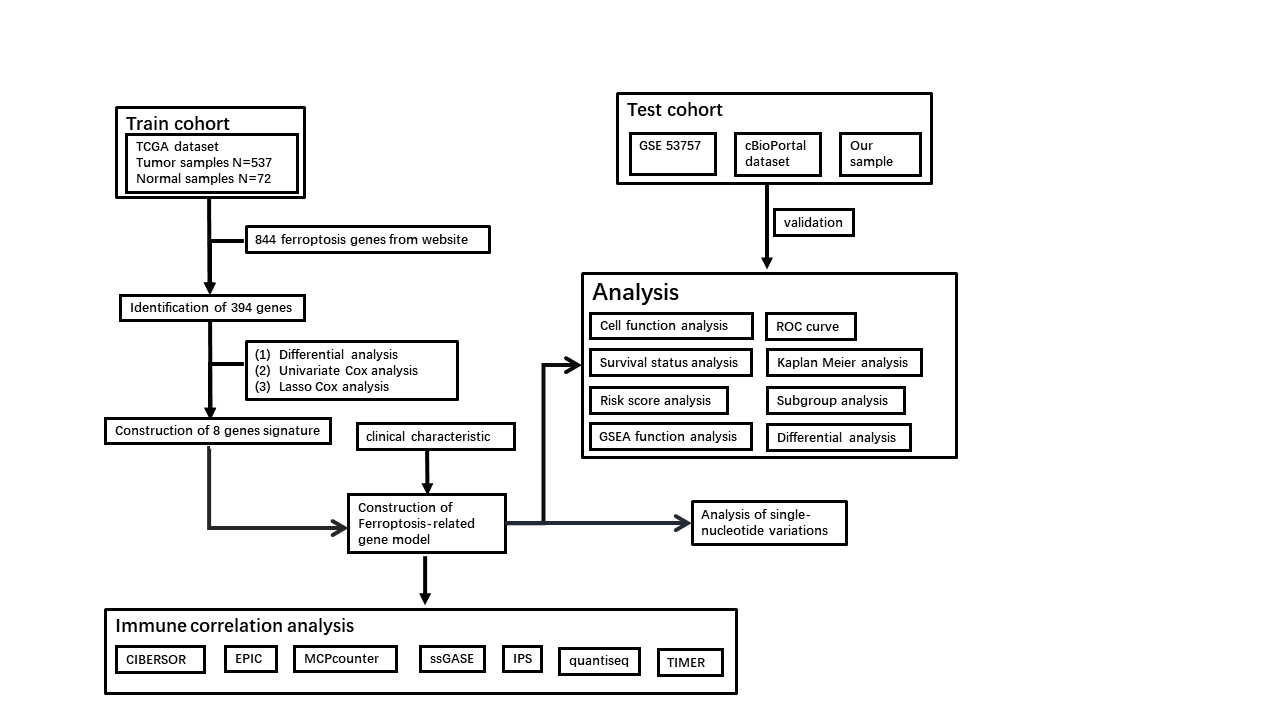

Supplement: Supplementary file 6 [file Image2.tif]

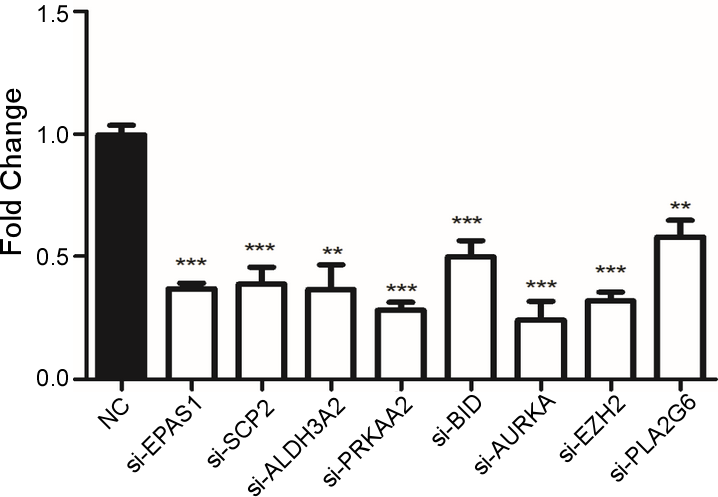

Supplement: Supplementary file 7 [file Image1.tif]

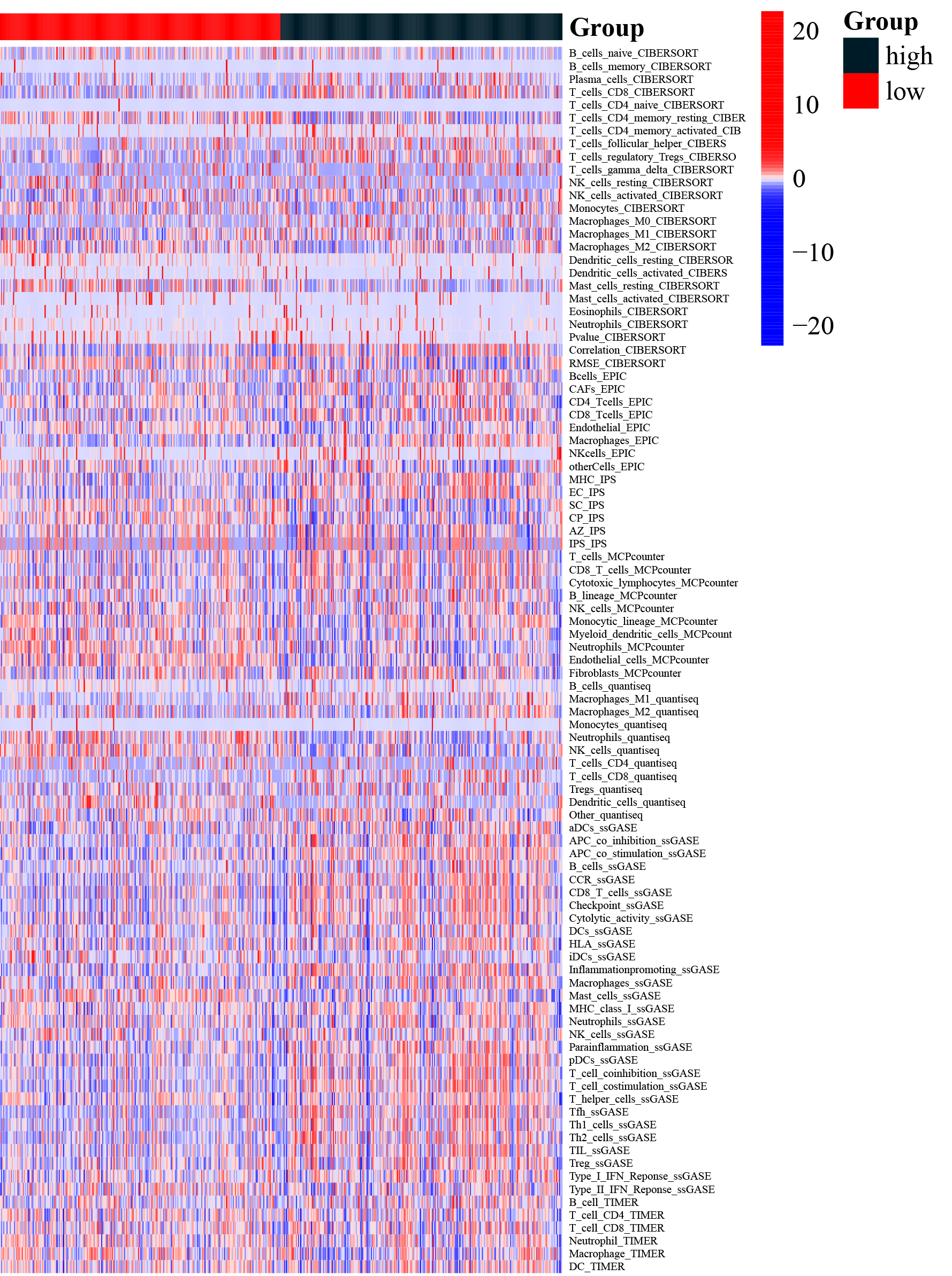

Supplement: Supplementary file 8 [file Image5.tif]
